# Extent of posterior wall lesion following pulmonary vein isolation with pentaspline pulsed field and cryoballoon ablation: the potential role of deployment technique

**DOI:** 10.3389/fcvm.2026.1880750

**Published:** 2026-10-07

**Authors:** Lucía Cobarro, Lorena Gómez-Burgueño, Martín Negreira-Caamaño, Isabel López-Alacid, Javier Ramos-Jiménez, Ez Alddin Rajjoub, Álvaro Marco-DelCastillo, Luis Borrego-Bernabé, Juan Delgado, Rafael Salguero-Bodes, Fernando Arribas-Ynsaurriaga, Daniel Rodríguez-Muñoz

**Affiliations:** 1 Department of Cardiology, Hospital Universitario 12 de Octubre. Avda. de Córdoba s/n, Madrid, Spain; 2 Research Institute Hospital Universitario 12 de Octubre (I+12), Madrid, Spain; 3 Department of Cardiology, Hospital Universitario de Getafe, Madrid, Spain; 4 Centro de Investigación Biomedica en Red Cardiovascular (CIBERCV), Madrid, Spain; 5 Faculty of Medicine, Complutense University of Madrid, Madrid, Spain

**Keywords:** ablation lesion assessment, pentaspline catheter, posterior wall isolated area, pulmonary vein isolation, pulsed field ablation

## Abstract

**Background:**

Pulmonary vein isolation (PVI) using cryoballoon (CB) or pulsed field ablation (PFA) with a pentaspline catheter achieves similar efficacy in atrial fibrillation (AF), but lesion extension on the left atrial posterior wall (LAPW) may differ. Pentaspline-PFA (P-PFA) has been reported to create broader LAPW lesions, potentially leaving narrow viable corridors that have been hypothesized to be proarrhythmic.

**Objective:**

To descriptively compare the extent of chronic unintended left atrial posterior wall isolation after PVI with P-PFA and CB ablation.

**Methods:**

We conducted a single-center ambispective study including consecutive patients undergoing repeat left atrial mapping after index PVI with either P-PFA, using a protocol designed to ensure catheter alignment with the pulmonary veins, or CB. Patients with additional LA ablation between index procedure and remapping, unrelated LAPW scarring, or posterior pulmonary vein gaps were excluded. The primary endpoint was isolated LAPW area. Secondary endpoints included percentage of isolated LAPW and minimum residual corridor width.

**Results:**

Seventy-four patients were included in the analysis: 49 P-PFA and 25 CB. Mean age was 64.2 ± 10.0 years, 64.9% were male, and 55.4% had persistent AF. Isolated LAPW area was 8.3 ± 4.0 cm^2^ in the P-PFA group and 9.5 ± 3.9 cm^2^ in the CB group, with no statistically significant difference detected between groups (mean difference −1.26 cm^2^, 95% CI: −3.22 to 0.70; *p* = 0.203). The percentage of isolated LAPW area was 39.9 ± 17.6% vs. 38.9 ± 14.9% (*p* = 0.805), and minimum residual corridor width was 19.7 ± 9.9 vs. 18.6 ± 11.0 mm (*p* = 0.671), respectively. P-PFA was associated with shorter procedure time (44.2 ± 10.2 vs. 65.8 ± 24.6 min; *p* < 0.001), shorter LA dwell time (38.1 ± 9.6 vs. 60.6 ± 20.5 min; *p* < 0.001), lower fluoroscopy time (5.3 ± 3.9 vs. 13.0 ± 9.7 min; *p* < 0.001), and lower dose–area product [5.3 (2.8–9.7) vs. 19.3 (6.5–31.0) Gy·cm^2^; *p* < 0.001].

**Conclusion:**

In this descriptive, hypothesis-generating study, no statistically significant difference in chronic LAPW isolation extent was detected between P-PFA and CB ablation. These findings support the hypothesis that catheter deployment technique may influence posterior wall lesion geometry, warranting confirmation in adequately powered prospective studies.

## Introduction

Pulmonary vein isolation (PVI) remains the cornerstone of catheter ablation for atrial fibrillation (AF) (1). Pulsed field ablation (PFA) has emerged as an effective alternative to thermal ablation, demonstrating comparable clinical efficacy in randomized and real-world studies (2–5). In addition, PFA exhibits a favorable safety profile due to reduced collateral damage to adjacent structures (6, 7).

The pentaspline PFA (P-PFA) catheter was designed to facilitate wide antral circumferential ablation. However, repeat procedures suggested that lesion sets created by this system may frequently involve the posterior wall (PW) of the left atrium (LA) to a substantial extent (8–10). This has been proposed to create a substrate for subsequent macroreentrant tachycardias or may result in inadvertent PW ablation (11). Conversely, several studies have suggested that more extensive posterior or antral lesion sets may be associated with reduced AF recurrence, either by directly modifying arrhythmogenic substrates or by serving as a surrogate for more extensive antral lesion sets (12, 13). This is consistent with the recognized role of the left atrial posterior wall (LAPW) in sustaining AF (14, 15), given its unique electrophysiological properties and frequent involvement during wide antral ablation strategies.

Understanding the long-term implications of this lesion geometry is critical, as it may influence both arrhythmia recurrence and procedural strategy. The P-PFA catheter generates a broad electric field that can extend beyond the intended antral target, potentially affecting adjacent PW myocardium. However, the acute extent of electrical silence may not necessarily reflect the final durable lesion set, because tissue at the periphery of the electric field may be exposed to sublethal field strengths, resulting in reversible or partially reversible electroporation (16, 17). This hinders the capacity of immediate post-ablation remapping to assess the extent of durably ablated PW, underscoring the need for delayed assessment to characterize definitive lesion extent (16, 18).

This descriptive, hypothesis-generating study aimed to compare the chronically ablated LAPW area following PVI using cryoballoon (CB) and P-PFA under a protocol designed to ensure catheter alignment with the pulmonary veins (PVs), without dedicated posterior anchoring.

## Methods

### Study patients

This was a single-center, ambispective observational study including consecutive patients with paroxysmal or persistent AF having undergone invasive LA remapping after an index PVI procedure with either P-PFA or CB. The prospective cohort was comprised of patients undergoing systematic remapping as part of a study to determine lesion characterization following a systematic workflow for PVI with the P-PFA catheter. The retrospective cohort included patients who had previously received index PVI using CB ablation. The inclusion criteria were 18 years of age or older, the ability to provide informed consent, and completion of repeat LA mapping with an electroanatomic navigation system. Patients were excluded if they had previous LA ablation beyond PVI, PW scarring unrelated to the index procedure, additional LA interventions between the index and remapping procedures, or non-isolated PV with conduction gaps located on the PW.

### Objective, primary and secondary endpoints

The objective of the study was to characterize the extent of LAPW isolation after P-PFA and to compare these findings with those observed after CB ablation. The primary endpoint was the posterior wall isolated area (PWIA). Based on previous reports, the posterior LA wall was anatomically defined as the quadrilateral area bounded superiorly by a line connecting the most cranial point of the ostia of the left and right superior PV, and inferiorly by a line connecting the most caudal point of the ostia of the left and right inferior PV. In patients with a common PV, the second branch of the common trunk was used as the anatomical reference for the ostium. Low-voltage regions were identified using thresholds consistent with previous studies, defined as <0.5 mV during sinus rhythm or atrial pacing, and <0.3 mV during atrial flutter or AF (11, 19, 20). Figure 1 shows the measurement details.

**Figure 1:**
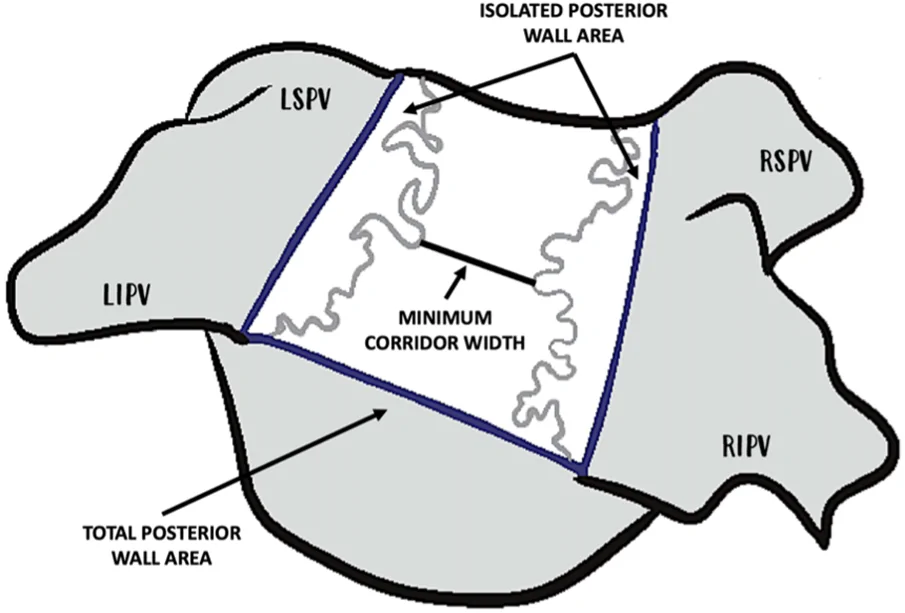
Schematic representation of the method used to define and quantify posterior wall lesion area. The total posterior wall surface was anatomically delineated as a quadrilateral area bordered superiorly by a line connecting the ostia of the left and right superior pulmonary veins and inferiorly by a line connecting the left and right inferior pulmonary veins. The ablated area was assessed as the region within this boundary that met low-voltage criteria (<0.5 mV in sinus rhythm or atrial pacing, <0.3 mV during flutter or atrial fibrillation). LIPV: left inferior pulmonary vein, LSPV: left superior pulmonary vein, RIPV: right inferior pulmonary vein, RSPV: right superior pulmonary vein.

Secondary endpoints included percentage of isolated LAPW area, minimum width of the residual corridor, fluoroscopy time, and safety outcomes. Major adverse events were defined as cardiac perforation, tamponade, stroke or transient ischemic attack, peripheral thromboembolic events, vascular complications requiring hospitalization or intervention, myocardial infarction, or death occurring within 30 days of either the index or remapping procedure.

### Periprocedural management

All patients underwent a transesophageal echocardiogram within 72 h prior to the procedure to rule out intracardiac thrombi. No preprocedural CT or intraprocedural intracardiac echocardiography was routinely performed. The procedure was performed under uninterrupted anticoagulation and either general anesthesia or deep sedation with propofol and fentanyl. Femoral vein punctures were performed under ultrasound guidance. Transseptal puncture (TSP) was carried out under fluoroscopic guidance. Unfractionated heparin was administered immediately after TSP targeting an activated clotting time target ≥ 300 s. Upon completion of the procedure, a figure-of-eight suture, and a pressure bandage were applied and removed four hours post-procedure. Oral anticoagulation was resumed 4 h after the ablation and continued for at least 2 months, or indefinitely based on thromboembolic risk, as assessed by either the CHA_2_DS_2_-VASc or CHA_2_DS_2_-VA score, as applicable. Antiarrhythmic drugs were not routinely prescribed, and any antiarrhythmic drug therapy was stopped 2 months post-ablation. As per protocol, patients were discharged 6 h after the procedure if no complications occurred.

Periprocedural management was the same across both groups.

### Index procedure ablation protocol

#### Cryoballoon ablation

Cryoballoon ablation was performed using the Arctic Front Advance Pro (Medtronic) or POLARx (Boston Scientific) systems. After TSP, a deflectable sheath was used to advance a 28-mm CB (Arctic Front Advance, Medtronic; or POLARx, Boston Scientific) into the LA. A spiral mapping catheter (Achieve, Medtronic; or Polarmap, Boston Scientific) was positioned in the target PV. Occlusion was confirmed with venography before a single 240-second freeze was applied per PV. Phrenic nerve pacing was performed from the superior vena cava to monitor nerve integrity throughout right-sided pulmonary vein CB ablation.

#### Pulsed field ablation

Atropine (1 mg) was administered after TSP to prevent vagal-induced bradycardia, except in patients with ventricular pacing devices. A 31 mm Farawave catheter (Boston Scientific, Marlborough, MA, USA) was advanced into the LA and navigated exclusively by fluoroscopic guidance following a fluoroscopy optimization protocol. This included use of single-frame or short-duration exposures using complementary projections—left anterior oblique (LAO) 30° and right anterior oblique (RAO) 20°, with an additional 20° caudal tilt for superior pulmonary veins—prior to each set of PFA applications to ensure contact, spline distribution and catheter-PV alignment. The catheter was deployed sequentially in basket and flower configurations to achieve complete ostial and antral coverage respectively, delivering at least eight pulse trains (2 kV/2.5 s, bipolar, biphasic) per configuration per vein. Reference fluoroscopic images of the initial catheter position were used to guide spline placement for subsequent applications. Fluoroscopy was repeated after each application to confirm catheter stability. If displacement was suspected or if more than 30 s elapsed between applications, the pair of applications was repeated. Additional applications were performed at the operator's discretion.

#### Mapping protocol and lesion measurement following CB ablation

High-density voltage mapping of the LA and PVs was performed using Pentaray (Biosense Webster), Orion (Boston Scientific), or HD Grid (Abbott) catheters. In the CB group, voltage mapping was performed immediately after transseptal access, at the beginning of redo procedures in patients with documented recurrence of AF or atrial tachycardia.

Patients with PV reconnection were excluded if residual conduction gaps were identified on the PW, which were systematically ablated using radiofrequency energy to achieve PVI. In those who met inclusion criteria, total PW area and non-isolated area were measured using previously validated voltage cut-offs (19) (amplitudes < 0.5 mV during sinus rhythm, <0.3 mV during atrial flutter and AF).

#### Mapping protocol and lesion measurement following P-PFA

In the P-PFA cohort systematic high-density electroanatomic remapping was performed immediately after TSP. Mapping was conducted using the same catheters and acquisition criteria as in the CB group. Patients with PV reconnection and residual conduction gaps on the PW were likewise excluded to avoid underestimation of lesion extent. LAPW area and the remaining electrically active surface were quantified using the same previously validated voltage thresholds.

### Statistical analysis

Continuous variables were expressed as mean ± standard deviation (SD) or median with interquartile range (IQR), according to data distribution. Normality was assessed using the Kolmogorov–Smirnov test. Categorical variables were reported as absolute numbers and percentages.

Comparisons between the P-PFA and CB groups were performed using the unpaired Student's *t*-test for normally distributed continuous variables and the Mann–Whitney *U* test for non-normally distributed continuous variables. Categorical variables were compared using the chi-square test or Fisher's exact test, as appropriate. Analyses were considered descriptive and exploratory; no noninferiority or equivalence framework was prespecified. A two-sided *p*-value < 0.05 was considered statistically significant.

Exploratory multivariable linear regression analyses were performed using PWIA as the primary dependent variable and ablation modality, LA size category, time to remapping, and mapping rhythm as covariates. Because heart failure could potentially influence voltage-based posterior wall measurements through atrial remodeling, fibrosis, and low-voltage substrate, a sensitivity multivariable model additionally including heart failure status was performed. Similar exploratory models were performed for percentage of isolated LAPW area and minimum residual corridor width.

All statistical analyses were performed using Stata IC version 16 (StataCorp LLC, College Station, TX, USA).

### Ethics approval

The study protocol was approved by the Research Ethics Committee for Medicines (CEIm) of Hospital Universitario 12 de Octubre (Madrid, Spain), within the framework of a larger study, and was conducted in accordance with the principles of the Declaration of Helsinki and current biomedical research regulations.

## Results

A total of 124 patients undergoing chronic remapping after index PVI with either CB or P-PFA were screened for eligibility, with 62 patients in each group. Overall, 37 patients in the CB group and 13 patients in the P-PFA group were excluded. Reasons for exclusion were additional LA ablation between the index procedure and remapping, most commonly ethanol infusion of the vein of Marshall, in 19 and 3 patients; posterior PV conduction gaps in 10 and 6 patients; and PW scarring unrelated to the index procedure in 8 and 4 patients, respectively (Figure 2).

**Figure 2:**
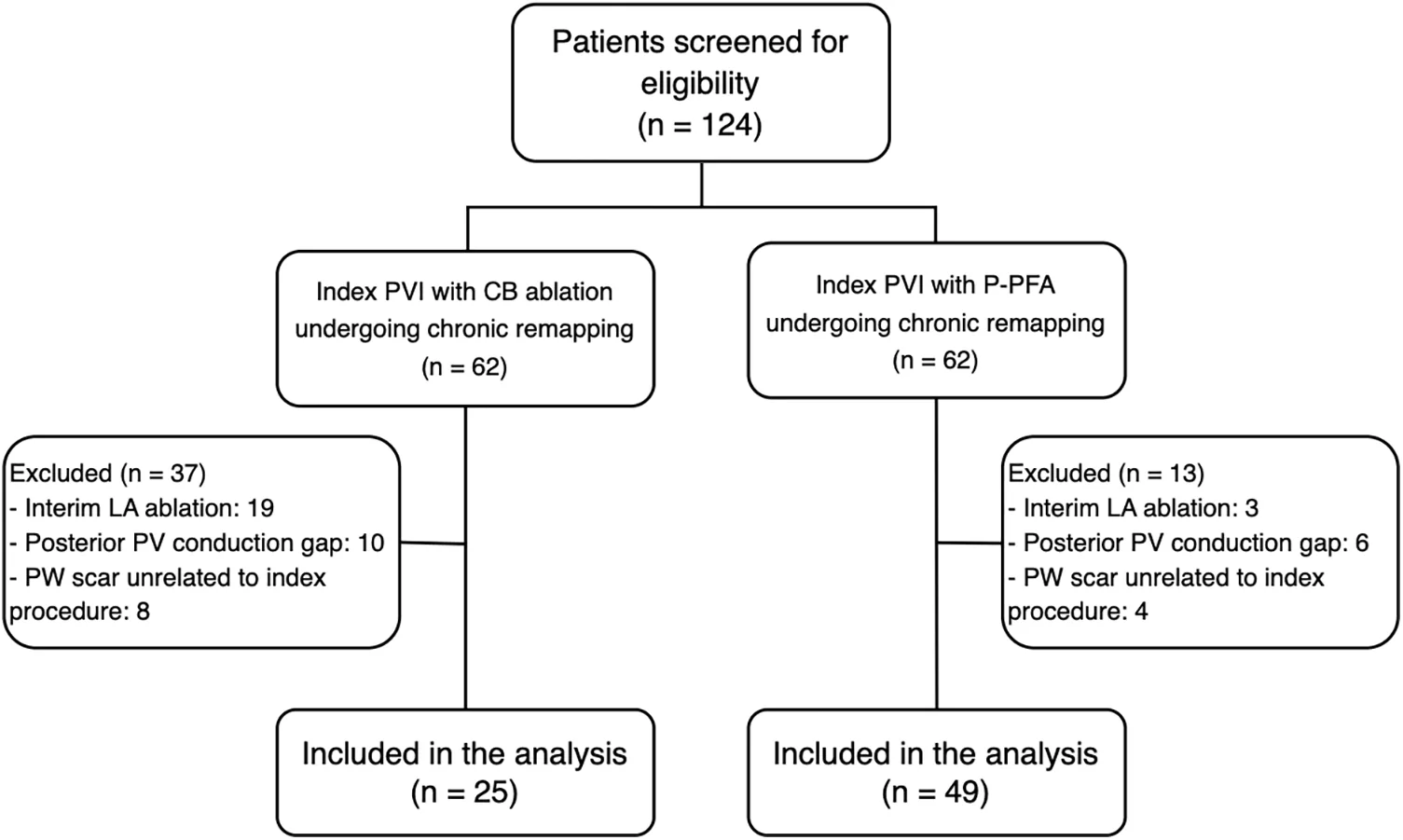
Patient flowchart. Patient screening, exclusion criteria, and final study population according to index ablation modality. Abbreviations as in text.

### Baseline characteristics

74 patients were included in the final analysis. Mean age was 64.2 ± 10.0 years, and 48 patients (64.9%) were male. Persistent AF was present in 41 patients (55.4%). The most prevalent comorbidities were hypertension (58.1%), dyslipidemia (37.8%), and diabetes mellitus (21.6%). Median left ventricular ejection fraction was 57% (IQR: 50–60). Baseline characteristics were generally balanced between groups, although heart failure was more frequent in the CB group and LA size distribution differed significantly between groups. Additional baseline characteristics are detailed in Table 1.

**Table 1:** Baseline characteristics of the study population. Values are mean ± SD, median (IQR), or *n* (%).

Variable	Overall	P-PFA	CB	*p* value
Age (yr.)	64.2 ± 9.98	65.6 ± 10.2	61.6 ± 9.1	0.104
Sex, male—*n* (%)	48 (64.9)	34 (69.4)	14 (56.0)	0.254
Hypertension—*n* (%)	43 (58.1)	30 (61.2)	13 (52.0)	0.447
Diabetes—*n* (%)	16 (21.6)	13 (26.5)	3 (12.0)	0.151
Dyslipidemia—*n* (%)	28 (37.8)	17 (34.7)	11 (44.0)	0.435
Body mass index (kg/m^2^)—mean ± SD	28.7 ± 4.16	29.2 ± 3.9	27.9 ± 4.5	0.239
Obstructive sleep apnea—*n* (%)	12 (16.2)	9 (18.4)	3 (12.0)	0.740
Previous stroke—*n* (%)	3 (4.0)	3 (6.1)	0 (0.0)	0.546
Heart failure—*n* (%)	13 (17.6)	5 (10.2)	8 (32.0)	0.027^a^
Chronic kidney disease—*n* (%)	4 (5.4)	3 (6.1)	1 (4.0)	0.546
CHADSVA—median (IQR)	2 (1–3)	2 (1–3)	1 (1–3)	0.315
Left Ventricular Ejection Fraction (%)—median (IQR)	57 (50–60)	58 (51–60)	57 (47–60)	0.660
Left Atrium Size				0.004^a^
Normal (≤34 mL/m^2^)	30 (40.5)	23 (46.9)	7 (28.0)	
Mildly dilated (35–41 mL/m^2^)	15 (20.3)	10 (20.4)	5 (20.0)	
Moderately dilated (42–48 mL/m^2^)	13 (17.6)	3 (6.1)	10 (40.0)	
Severely dilated (>48 mL/m^2^)	16 (21.6)	13 (26.5)	3 (12.0)	
Persistent atrial fibrillation—*n* (%)	41 (55.4)	27 (55.1)	14 (56.0)	0.941
Antiarrhythmic drug treatment				0.252
Amiodarone—*n* (%)	7 (9.5)	3 (6.1)	4 (16.0)	
Flecainide—*n* (%)	9 (12.2)	5 (10.2)	4 (16.0)	
Oral anticoagulation				0.072
Vitamin K antagonist—*n* (%)	9 (12.2)	8 (16.3)	1 (4.0)	
Direct oral anticoagulant—*n* (%)	61 (82.4)	40 (81.6)	21 (84.0)	

*P* values compare P-PFA vs CB. For categorical variables with subcategories, the *p* value refers to the overall distribution. CB, cryoballoon; P-PFA, pentaspline pulsed field ablation.

a Statistically significant difference between groups, *p* < 0.05.

### Index procedure

Index PVI was performed using CB in 25 patients and P-PFA in 49 patients.

The median time between AF diagnosis and the first ablation procedure was 358 days (IQR: 139–1,336). The mean total procedure time was 65.8 ± 24.6 min in the CB group and 44.2 ± 10.2 min in the PFA group. The mean LA dwell time was 60.6 ± 20.5 and 38.1 ± 9.6 min, respectively. Both total procedure time and LA dwell time were significantly shorter with PFA compared to CB (*p* < 0.001 for both comparisons).

All procedures were performed in overlapping time periods to minimize temporal biases.

### Mapping procedure following CB ablation

In patients included in the CB cohort, the median time from the index procedure to remapping was 880 days (IQR: 564–1,306). Durable PVI was confirmed in 14 patients (56.0%). Voltage mapping was performed during sinus rhythm or atrial pacing in 21 (84.0%) patients, while the rest were mapped in AF. In 17 patients (68.0%), no low-voltage areas were observed in the LA outside the isolated PVs.

### Mapping procedure following P-PFA

Among patients in the PFA cohort, the median time from the index procedure to remapping was 76 days (IQR: 38–170).

Remapping was performed due to arrhythmic recurrence in four patients: two with AF and two with left atrial flutter. Voltage mapping was performed in AF or left atrial flutter, respectively, in these patients. In one AF patient, reconnection of the left inferior PV due to an anterior gap was identified, whereas the remaining three had durably isolated PVs. Neither of the two patients presenting with AF showed low-voltage areas in the LA.

One patient with left atrial flutter demonstrated a double-loop macroreentrant circuit, consisting of a clockwise perimitral flutter and a second loop around the left PVs with a shared isthmus. An anterior low-voltage scar was identified. The arrhythmia was successfully treated by achieving bidirectional mitral isthmus block, which required ethanol infusion into the vein of Marshall. The other patient had a typical perimitral flutter, successfully treated with an anterior mitral line, and showed no low-voltage areas in the LA.

In the remaining 45 patients, remapping was performed as part of the systematic follow-up protocol, and voltage mapping was conducted during sinus rhythm or atrial pacing in all cases. Durable PVI was confirmed in 39 of 45 patients (86.7%). The LA was electrically healthy in 42 patients (93.3%).

### Isolated area on posterior wall

The median number of electrograms recorded with the mapping catheter was higher in the P-PFA group than in the CB group: 11,497 (IQR: 7,620–18,739) vs. 8,126 (IQR: 4,079–11,477), respectively (*p* = 0.015).

The mean PWIA was 8.3 ± 4.0 cm^2^ in the P-PFA group, 9.5 ± 3.9 cm^2^ in the CB group, with no statistically significant difference detected between groups (mean difference −1.26 cm^2^, 95% CI: −3.22 to 0.70; *p* = 0.203). The percentage of isolated LAPW area was 39.9 ± 17.6% in the P-PFA group and 38.9 ± 14.9% in the CB group, with no statistically significant difference detected between groups (mean difference 1.02 percentage points, 95% CI: −7.18 to 9.22; *p* = 0.805). Total LAPW area was significantly smaller in the P-PFA group than in the CB group (20.9 ± 5.0 vs. 24.9 ± 5.1 cm^2^; *p* = 0.002), as was the non-isolated PW area (12.6 ± 5.1 vs. 15.4 ± 5.3 cm^2^; *p* = 0.032). Similar results were obtained in the subgroup of patients mapped during sinus rhythm or atrial pacing.

Quantitative PW metrics and procedural parameters according to ablation modality are summarized in Table 2. Representative examples of lesion sets are shown in Figures 3, 4.

**Table 2:** Quantitative assessment of posterior wall isolation and procedural characteristics stratified by ablation technology.

Variable	P-PFA (*n* = 49)	CB (*n* = 25)	*p*-value
Isolated PW area, cm^2^ (mean ± SD)	8.3 ± 4.0	9.5 ± 3.9	0.203
Percentage of isolated PW area, % (mean ± SD)	39.9 ± 17.6	38.9 ± 14.9	0.805
Total PW area, cm^2^ (mean ± SD)	20.9 ± 5.0	24.9 ± 5.1	0.002^a^
Non-isolated PW area, cm^2^ (mean ± SD)	12.6 ± 5.1	15.4 ± 5.3	0.032^a^
Minimum corridor width, mm (mean ± SD)	19.7 ± 9.9	18.6 ± 11.0	0.671
Minimum corridor width < 10 mm, *n* (%)	10 (20.4)	6 (24.0)	0.770
Minimum corridor width <15 mm, *n* (%)	18 (36.7)	10 (40.0)	0.805
Fluoroscopy time, min (mean ± SD)	5.3 ± 3.9	13.0 ± 9.7	<0.001
Dose–area product (median, IQR)	5.3 (2.8–9.7)	19.3 (6.5–31)	<0.001
Left atrial dwell time, min (±SD)	38.1 (±9.6)	60.6 (±20.5)	<0.001
Procedure time, min (±SD)	44.2 (±10.2)	65.8 (±24.6)	<0.001
No. of points, *n* (IQR)	11,497 (7,620–18,739)	8,126 (4,079–11,477)	0.015

Abbreviations as in text.

a Statistically significant difference between groups, *p* < 0.05.

**Figure 3:**
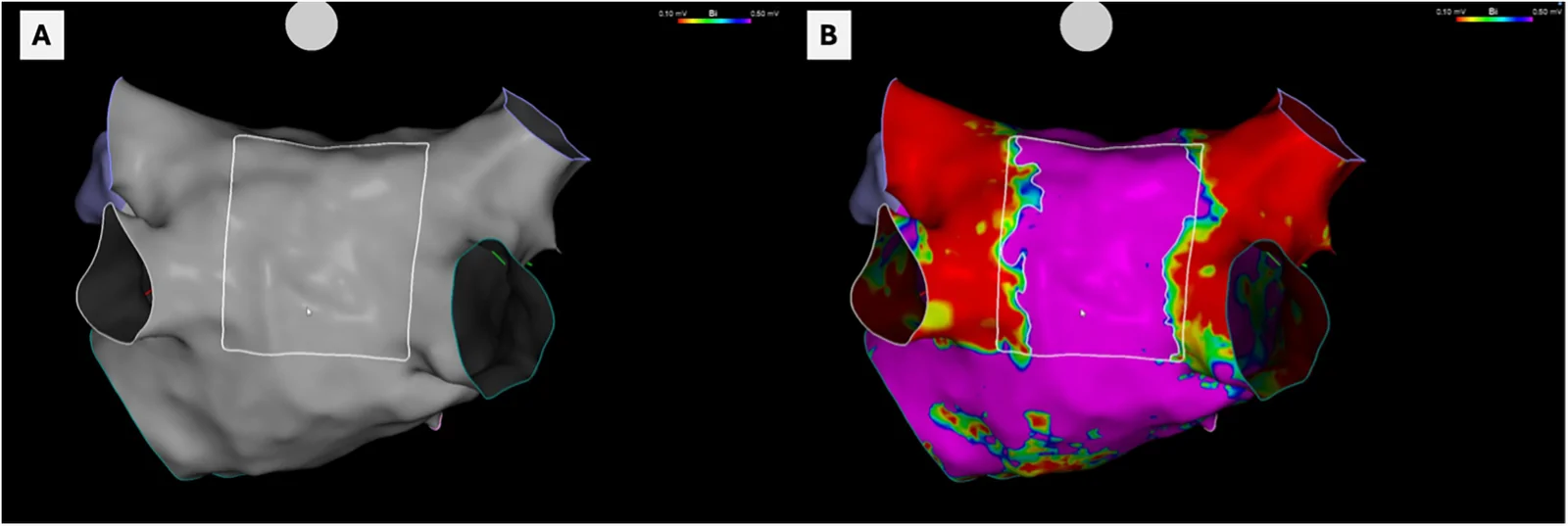
Assessment of posterior wall isolation using the CARTO3 (Biosense Webster, Irvine, CA, USA) mapping system after cryoballoon ablation. **(A)** Three-dimensional electroanatomical reconstruction of the left atrium in sinus rhythm, showing the manually defined anatomical boundaries of the posterior wall. **(B)** Bipolar voltage map acquired in sinus rhythm with a threshold of ≥0.5 mV to identify healthy myocardium (purple), illustrating the manually delineated area to quantify the isolated region after ablation.

**Figure 4:**
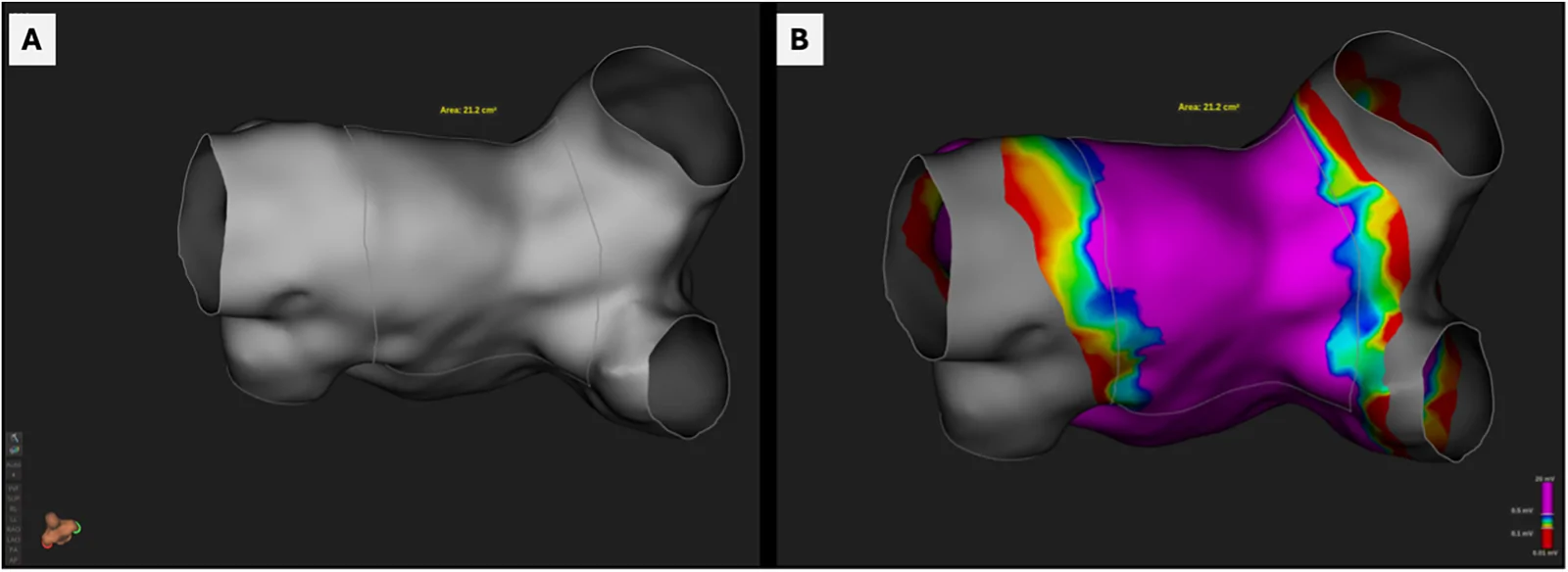
Assessment of posterior wall isolation using the RHYTHMIA^TM^ system after pulsed field ablation for pulmonary vein isolation. **(A)** High-density electroanatomical map of the left atrium acquired in sinus rhythm following pulmonary vein isolation with PFA. **(B)** Bipolar voltage map using a ≥0.5 mV threshold to define healthy myocardium (purple), with manual delineation of the isolated posterior wall area to quantify the extent of lesion formation.

### Minimum corridor width

Minimum corridor width was 19.7 ± 9.9 mm in the P-PFA group and 18.6 ± 11.0 mm in the CB group, with no statistically significant difference detected between groups (*p* = 0.671). Findings were consistent in the subgroup mapped during sinus rhythm or atrial pacing (20.1 ± 9.9 vs. 19.2 ± 11.9 mm; *p* = 0.752).

Complete fusion of posterior PV lesions precluded measurement of any residual PW corridor in three patients (two in the CB group, one in the PFA group).

When corridor width was analyzed as a dichotomous variable, 10 patients (20.4%) in the P-PFA group had a minimum PW corridor width < 10 mm, compared with 6 patients (24.0%) in the CB group, with no significant difference (*p* = 0.770). Using a 15 mm threshold, 18 of 49 patients (36.7%) in the P-PFA group and 10 of 25 patients (40.0%) in the CB group had a minimum corridor width < 15 mm, again with no significant difference (*p* = 0.805).

### Exploratory multivariable and sensitivity analyses

Because LA size, time to remapping, and mapping rhythm may influence voltage-based estimates of PW lesion extent, an exploratory multivariable linear regression analysis was performed for the primary endpoint. The model included ablation modality, LA size category, time from index ablation to remapping, and mapping rhythm. After adjustment, ablation modality was not independently associated with PWIA. The adjusted difference in PWIA for CB compared with P-PFA was 0.94 cm^2^ (95% CI: −1.70 to 3.59; *p* = 0.478). LA size and time to remapping were not significantly associated with PWIA. Mapping during AF, however, was associated with larger measured PWIA compared with sinus rhythm or atrial pacing, with an adjusted difference of 3.11 cm^2^ (95% CI: 0.91 to 5.31; *p* = 0.006).

Additional exploratory multivariable models were performed for the secondary posterior wall metrics using the same covariates. After adjustment, ablation modality was not significantly associated with percentage of isolated LAPW area or minimum residual corridor width.

In the sensitivity multivariable model including heart failure status, heart failure was not significantly associated with PWIA (adjusted difference −0.07 cm^2^; 95% CI: −2.46 to 2.33; *p* = 0.956), and ablation modality remained non-significant. Heart failure was also not significantly associated with percentage of isolated LAPW area or minimum residual corridor width.

Propensity score matching including clinical characteristics and available anatomical variables yielded 15 matched pairs. After matching, total PW area was well balanced between P-PFA and CB (23.31 vs. 23.37 cm^2^; SMD = 0.01). The primary findings remained unchanged, with no significant differences in PWIA (9.76 ± 4.67 vs. 9.68 ± 3.69 cm^2^; *p* = 0.958), percentage of isolated LAPW (42.64 ± 21.09% vs. 42.20 ± 14.70%; *p* = 0.945), or minimum residual corridor width (21.23 ± 9.96 vs. 17.22 ± 12.32 mm; *p* = 0.403).

### Macroreentrant atrial tachycardia

After a median follow-up of 367 days (IQR, 179–786 days), left atrial macroreentrant tachycardia involving the PW was observed in two patients in the CB group (8.0%) (median follow-up 1,453 days, IQR: 1,261–1,770), and three patients in the PFA group (6.1%) (median follow-up 296, IQR: 130–420) (*p* = 0.82). This comparison should be interpreted with caution given the low number of events and the heterogeneous follow-up duration among groups. Additionally, non–PW–dependent left atrial macroreentrant tachycardia occurred in two patients treated with CB ablation and in one patient treated with PFA.

Among the five patients who developed PW–dependent macroreentrant tachycardia, two patients in the CB group (9.3 mm and 14.6 mm) and one in the PFA group (10.3 mm) had a minimum PW corridor width < 15 mm.

### Fluoroscopy outcomes

Mean fluoroscopy time was significantly shorter in the P-PFA group than in the CB group (5.3 ± 3.9 vs. 13.0 ± 9.7 min; *p* < 0.001). Radiation exposure, expressed as dose–area product, was also significantly lower with P-PFA than with CB ablation [5.3 (IQR: 2.8–9.7) vs. 19.3 (IQR: 6.5–31.0) Gy·cm^2^; *p* < 0.001].

### Safety outcomes

One patient in the CB group experienced a vascular complication following the index procedure, requiring extended hospitalization for observation, although no further intervention was needed. No complications related to the index procedure were observed in the P-PFA group. However, one patient who underwent systematic remapping developed acute decompensated heart failure requiring hospital admission and intravenous furosemide. No other complications were observed, including no cases of stroke or transient ischemic attack, peripheral thromboembolic events, vascular complications requiring intervention, myocardial infarction, or death within 30 days following either the index or remapping procedure.

## Discussion

The extent of PWIA following PVI achieved with the P-PFA catheter has attracted considerable attention since early clinical experiences. Both acute (20) and chronic (11, 16) remapping studies have reported broad lesion sets involving the LAPW, raising concerns regarding unintended substrate modification. Additionally, some reports have linked narrow residual viable corridors within the LAPW to the development of macroreentrant atrial tachycardias in patients undergoing P-PFA (11). Critically, most studies reporting wide LAPW isolation, and subsequent macroreentrant atrial tachycardia circuits have originated from early experiences with PFA, when lesion delivery protocols often included systematic posterior anchoring to ensure antral coverage. This technique may have led to unintended more extensive LAPW lesions (18). These observations raise the possibility that lesion extent may not be a fixed property of the P-PFA system itself, but rather a modifiable outcome determined by ablation technique. In addition, acute remapping may overestimate the extent of durable lesions in PFA, particularly at the periphery of the electric field. Therefore, chronic-phase remapping (beyond 30 days) is essential for accurately assessing lesion integrity (17, 21).

In this context, our study offers meaningful contributions. First, remapping was systematically performed at least 30 days after the index procedure, a time frame that more reliably reflects durable lesion formation and minimizes the overestimation associated with acute-phase assessments. Second, all procedures adhered to a standardized workflow that avoids routine posterior anchoring. Instead, applications were delivered in catheter positions carefully confirmed under orthogonal fluoroscopic views (LAO 30°, RAO 20°), aiming for alignment with the PV trajectory. This approach aligns with current best practices (22) and contrasts with earlier techniques that may have unintentionally promoted PW debulking. Third, we included a contemporary CB cohort as a comparator, providing context for the interpretation of PWIA after P-PFA.

In our cohort, no statistically significant difference in LAPW lesion extent was detected between P-PFA and CB ablation, although these findings should not be interpreted as evidence of equivalence or noninferiority. The mean isolated LAPW area after P-PFA was 8.3 ± 4.0 cm^2^, corresponding to 39.9 ± 17.6% of the LAPW. Our findings contrast with those reported by Bohnen et al. (20), who described an acutely isolated posterior wall area of 13 cm^2^ following PFA—a value that may, in part, reflect reversible electroporation or transient electrical stunning, potentially overestimating the extent of chronic durable lesion formation. Similarly, Kawamura et al. (16) reported a mean chronic isolated LAPW area of 11 cm^2^, corresponding to approximately 50% of the total PW surface. In comparison, the mean isolated area in our P-PFA cohort was smaller, suggesting a more confined chronic lesion profile despite use of the same catheter under a different deployment strategy. Nevertheless, the confidence interval for the between-group difference in PWIA ranged from −3.22 to 0.70 cm^2^, indicating that potentially clinically meaningful differences cannot be excluded, particularly because no validated threshold defines a clinically relevant PWIA difference.

Taken together, our findings challenge the prevailing notion that the P-PFA catheter inherently produces extensive PW ablation. Instead, they support the hypothesis that LAPW lesion extent after P-PFA may be influenced by procedural technique rather than being solely determined by the ablation modality or catheter design.

From a clinical perspective, this has two potential implications: first, the presence of incidental PWIA should not be considered a definitive argument for or against the use of P-PFA relative to other technologies. Second, strategies aimed at empirically completing LAPW isolation to avoid narrow viable corridors—particularly when based on acute remapping data or early studies using routine posterior anchoring—may need to be re-evaluated in light of contemporary optimized PFA workflows (22). Integration of PFA catheters with 3D-EAM systems may improve lesion assessment and understanding of energy delivery sites (23, 24).

An additional focus of our analysis was the minimum corridor width in the LAPW, which may represent a relevant anatomical parameter. Although narrow residual isthmuses in the LAPW have been implicated in the development of macroreentrant tachycardias, no specific anatomical cut-off value has been formally validated. Katritsis et al. described a mean anatomical corridor width of 8.9 ± 3.5 mm in patients with scar-related perimitral flutters (25). In the absence of validated anatomical thresholds, we selected 10 mm and 15 mm as descriptive reference points to characterize residual corridors, based on ranges reported in prior studies (25) and to provide reproducible descriptive reference points.

Furthermore, recent LGE-CMR data have shown that lesion geometry differs across ablation technologies, with PFA and CB producing wider peri-PV lesions than conventional radiofrequency ablation. However, LGE-CMR primarily characterizes anatomical lesion width and continuity around the PVs, and whether these findings translate into greater functional and durable LAPW isolation remains uncertain (26). Our study extends these observations by using invasive high-density electroanatomic remapping to quantify chronic LAPW isolation after P-PFA and CB ablation.

Finally, our center has implemented a low-fluoroscopy protocol for PFA procedures, as described in the Methods section. This strategy enabled a statistically significant reduction in radiation exposure in the P-PFA cohort. The notably shorter fluoroscopy times observed in the PFA group reflect the effectiveness of this workflow and highlight the potential of PFA to further optimize procedural safety.

### Limitations

Our study has limitations, mainly due to its ambispective and observational nature. First, the choice of ablation modality reflected institutional workflow rather than randomization. Systematic remapping was performed in all P-PFA patients as part of a research protocol, whereas in the CB group remapping was limited to patients with clinical indications for a redo procedure. In addition, predefined exclusion criteria, particularly interim LA ablation before remapping and posterior PV conduction gaps, affected the two cohorts differently and reduced the number of eligible patients, precluding a matched cohort design. The substantially different interval between index ablation and remapping in the two cohorts represents an additional potential source of confounding, although time to remapping was not significantly associated with PWIA in the adjusted analysis.

Second, the use of different navigation systems and modules contributed to data heterogeneity. In particular, the higher point density observed in the PFA group is likely related to the use of the Orion catheter. Third, differences in mapping rhythm might have influenced the reported extent of ablated areas (20, 27, 28). Sinus rhythm or atrial pacing is generally preferred for standardized voltage assessment, but some remapping procedures were performed during AF or atrial flutter to allow comprehensive clinical management. However, results observed in the subgroup of patients mapped in sinus rhythm were consistent with those of the overall cohort, and rhythm-specific low-voltage thresholds were applied. Fourth, baseline imbalances in LA size and heart failure prevalence may have influenced voltage-based PWIA measurements. Although adjusted exploratory analyses yielded consistent findings, residual confounding cannot be excluded. Also, individual PV dimensions were not systematically available and could not be accounted for in the adjusted analyses; therefore, residual confounding related to PV anatomy cannot be excluded. These limitations highlight the need for prospective confirmation in more balanced populations using standardized assessment across ablation strategies. Fifth, this study reflects the experience of a high-volume teaching center using a standardized low-fluoroscopy P-PFA workflow. Therefore, procedural time, fluoroscopy exposure, and lesion geometry may not be fully generalizable. Finally, a major limitation of our study is that it was not designed or powered to establish equivalence or noninferiority between ablation modalities. *Post-hoc* power estimation confirmed limited statistical power for the primary endpoint, mainly due to the small CB group and observed PWIA variability; therefore, smaller but potentially clinically meaningful differences cannot be excluded.

## Conclusion

In this descriptive, hypothesis-generating study, no statistically significant difference in chronic LAPW lesion extent was detected between P-PFA using a catheter-alignment workflow and CB ablation. Alignment between catheter and PV and avoidance of systematic posterior anchoring may prevent the generation of potentially proarrhythmic chronic narrow corridors. These findings support the hypothesis that catheter deployment technique may influence posterior wall lesion geometry and warrant confirmation in adequately powered prospective studies.

## Data Availability

The raw data supporting the conclusions of this article will be made available by the authors, without undue reservation.
